# The effects of long-term almond consumption on whole-body insulin sensitivity, postprandial glucose responses, and 48 h continuous glucose concentrations in males and females with prediabetes: a randomized controlled trial

**DOI:** 10.1007/s00394-023-03178-w

**Published:** 2023-05-31

**Authors:** Elske Gravesteijn, Ronald P. Mensink, Jogchum Plat

**Affiliations:** Department of Nutrition and Movement Sciences, NUTRIM School of Nutrition and Translational Research in Metabolism, Maastricht University Medical Center+ (MUMC+), P.O. Box 616, 6200 MD Maastricht, The Netherlands

**Keywords:** Almonds, Prediabetes, Insulin sensitivity, Glucose metabolism, Hyperinsulinemic euglycemic clamp, Human intervention

## Abstract

**Purpose:**

Findings concerning the effects of almond consumption on glucose metabolism are inconsistent which might relate to body weight gain. The effects of long-term almond consumption on glucose metabolism are investigated in a free-living setting without detailed dietary instructions in males and females with overweight/obesity and prediabetes.

**Methods:**

Forty-three participants volunteered in this randomized, cross-over trial with a 5-months control and intervention period and a 2-months wash-out. In the intervention period participants daily consumed 50 g whole almonds. At the end of both periods insulin sensitivity was assessed by a hyperinsulinemic euglycemic clamp, and postprandial glucose responses, and 48 h continuous glucose concentrations were measured.

**Results:**

Almond consumption significantly decreased insulin sensitivity (P = 0.002), and increased postprandial glucose concentrations (P = 0.019), as well as fasting insulin concentrations (P = 0.003) as compared to the control period. The AUCs for 24 h glucose concentrations were not significantly different between control and intervention (P = 0.066). Almond consumption also significantly increased BMI (P = 0.002), and waist circumference (P = 0.013), supported by the concurrent increased energy intake (P = 0.031). The effects on glucose metabolism could only partly be explained by the observed weight gain as the almond effect remained after correcting for BMI changes.

**Conclusions:**

In participants with prediabetes, long-term almond consumption showed adverse effects on insulin sensitivity and glucose metabolism. As almonds seemed not to have fully replaced other food items, it might be necessary to provide more supporting guidelines on how to incorporate energy-dense nuts into healthy diets to prevent type 2 diabetes development.

**Clinical Trial Registration:**

This clinical trial was registered in February 2018 as NCT03419702.

## Introduction

The prevalence of prediabetes is increasing worldwide [1], which is alarming for healthcare since it is highly associated with an increased risk of developing type 2 diabetes [2] and cardiovascular disease [3]. Prediabetes is an intermediate metabolic condition that is characterized by reduced insulin sensitivity leading to temporary glucose excursions [4], but not yet as pronounced as in type 2 diabetes [5]. Either fasting plasma glucose concentrations are increased and/or postprandial glucose tolerance is impaired. Several lifestyle interventions have been shown to mitigate the risk of prediabetes developing into type 2 diabetes by improving insulin sensitivity and consequently normalizing glucose regulation [2].

Effective lifestyle interventions that have been linked to reversing prediabetic conditions and its consequences are weight loss, increased physical activity, and consuming a healthy diet or specific foods [6]. For example, nut consumption as part of a healthy diet is associated with a decreased risk of cardiovascular disease and type 2 diabetes as compared to adapting a healthy diet alone [7]. This effect can most likely be attributed to the fact that nuts are rich in nutrients and bioactive compounds such as unsaturated fatty acids, L-arginine, fibers, minerals, vitamins, plant sterols, and polyphenols. There are, however, slight differences in composition between nuts [8]. In comparison to other nuts, almonds are particularly rich in protein, fiber, riboflavin, niacin, ɑ-tocopherol, and calcium that could contribute to improving a disturbed glucose metabolism [9]. A number of studies already showed beneficial effects of almonds in both patients with prediabetes and type 2 diabetes. Patients with type 2 diabetes who consumed 60 g of almonds per day as a 20% caloric replacement from a control National Cholesterol Education Program step II diet for 4 weeks showed decreased fasting glucose and insulin concentrations, and improved insulin sensitivity as assessed by the homeostatic model assessment for insulin resistance (HOMA-IR; [10]). Similar effects on glycemic control were found in individuals with prediabetes after consuming 57 g almonds for 16 weeks [11]. Besides these effects on fasting glycemia after 4 to 16 weeks almond consumption, there are also acute effects of almond consumption in healthy individuals showing decreased postprandial glucose and insulin responses after a test meal with 60 g almonds [12]. However, results are inconsistent since not all studies evaluating the effects of almonds showed these beneficial effects. For example, Palacios et al*.* [13] found no effects upon consuming 43 g almonds per day for 6 weeks on glucose and insulin concentrations, and insulin sensitivity indices such as insulin sensitivity index (Si), acute insulin response to glucose (AIRg), pancreatic beta-cell function measured as disposition index (DI), and fasting homeostasis model assessments of beta-cell function (HOMA2-%B) in adults with prediabetes. This finding was in line with observations by Madan et al*.* [14] who reported no changes in glucose and insulin concentrations, and HOMA-IR in adolescents with prediabetes consuming 56 g almonds per day for 12 weeks. The question is how these discrepant findings can be explained. In general, it seems that the health effects of almonds are evident in both long-term [11], and shorter-term intervention studies [10], with a comparable daily intake of almonds as the studies that did not show an effect [13–15]. Therefore, it is not likely that the inconsistent results can be attributed to the amounts of almonds consumed daily or to study duration. In most controlled trials, however, almonds were iso-calorically added to the diet replacing a certain percentage of daily energy intake from other food sources. When no weight changes were observed, several of these studies found a beneficial almond effect on insulin and HOMA-IR [11], whereas other studies did not find changes in markers of glucose metabolism such as glucose, insulin, and HOMA-IR [13–15]. Despite a study design with iso-caloric substitution, in one study body fat reduced in conjunction with a beneficial almond effect on glucose, insulin, and HOMA-IR [10], whereas in another study participants gained body weight in the absence of an almond effect on insulin sensitivity or glycemia [16]. Therefore, it is possible that the absence or presence of an almond effect relates to changes in body weight. We therefore investigated the effects of almonds without detailed dietary instructions or adjustments for body weight. However, weight stability was monitored and in case of weight fluctuations the participant received an adjusted dietary advice in order to try to correct this fluctuation.

It should also be considered that the improvements found in insulin resistance upon almond consumption so far are based on improvements of HOMA-IR. Although HOMA-IR is frequently used and is an established risk marker that is also used in clinical practice [17], it is a surrogate marker whereas calculating the M-value based on a hyperinsulinemic euglycemic clamp is the actual gold standard to assess insulin sensitivity [18]. As both the hyperinsulinemic euglycemic clamp and the postprandial test are performed under well-controlled lab settings, it is also relevant to examine effects of almond consumption on glucose metabolism under free-living conditions. Therefore, we here examined the effects of long-term almond consumption in males and females with prediabetes on glucose metabolism side-by-side via the clamp technique, in the postprandial state, and in real life conditions using continuous glucose monitors.

## Methods

### Participants

Forty-three males and females between 40 and 75 years with prediabetes were recruited via advertisements and posters in local newspapers, university and city buildings, hospital, public transport, and online. Prediabetes was defined as either having impaired fasting glucose (IFG; fasting glucose 6.1–6.9 mmol/L) and/or impaired glucose tolerance (IGT; 2-h glucose 7.8–11.0 mmol/L), according to the WHO criteria [19]. This was assessed with a 75 g oral glucose tolerance test (OGTT) during a screening visit at the research facilities in the Metabolic Research Unit Maastricht (MRUM) after a 12-h overnight fast. Fasting blood samples were also analyzed for serum total cholesterol (TC), triacylglycerol (TAG), and potassium (K), and for EDTA plasma haemoglobin (Hb). In case the OGTT indicated that volunteers indeed suffered from prediabetes (IGT, or IFG and IGT combined) or when fasting glucose concentrations were increased, a second screening visit was planned to take another fasting blood sample to ensure that the average glucose, TC, and TAG concentrations of both screening visits met the criteria of TC < 8.0 mmol/L, TAG < 4.52 mmol/L, K 3.60–5.00 mmol/L, and Hb 8.2–11.0 mmol/L (men) or 7.3–9.7 mmol/L (females).

Eligibility was further evaluated based on the following inclusion criteria: body mass index (BMI) 25–40 kg/m^2^; stable body weight (weight gain or loss < 3 kg in the past three months); nonsmoker or smoking cessation > 1 year; no diabetes; no familial hypercholesterolemia; no abuse of drugs; not > 4 alcoholic consumptions per day with a maximum of 21 per week; no use of medication to treat blood pressure, lipid or glucose metabolism; no severe medical conditions that might interfere with the study such as epilepsy, asthma, kidney failure or renal insufficiency, chronic obstructive pulmonary disease, inflammatory bowel diseases, auto inflammatory diseases, or rheumatoid arthritis; no active cardiovascular diseases or events such as congestive heart failure, acute myocardial infarction, or cerebrovascular accident; no allergy or intolerance to almonds. Before the first screening visit, all volunteers signed informed consent. Study performance was in accordance with the ethical guidelines of the Declaration of Helsinki. The study was approved by the Medical Ethical Committee of the University Hospital Maastricht/Maastricht University (METC azM/UM; METC173015), registered in February 2018 at ClinicalTrials.gov (NCT03419702), and conducted between February 2018 and December 2021.

### Study design

This 1-year intervention study had a randomized, controlled, cross-over design with an intervention and control period of both 5 months, separated by a wash-out period of 2 months. Randomization into 8 blocks was performed by an independent researcher using a computer-generated randomized block design with stratification for gender. During the intervention period participants daily consumed 50 g of whole, unsalted, unroasted almonds (Almond Board of California, California, United States). The nuts could be consumed at any time of the day, but all at once in one portion and not spread over the day. There were no instructions that the almonds should be consumed instead of any other item in the diet, allowing participants decide themselves as such mimicking the general guidelines to incorporate a handful of nuts in the daily diet. It was not allowed to consume any other nuts or nut products. Participants received a list of products that were not allowed at the start of the study and this limitation was emphasized during the follow-up visits. Participants were asked to note down their almond consumption in a diary, and to return the used and unused sachets to monitor compliance. During the control period participants were not allowed to eat almonds or any other nuts or nut products. All participants received instructions how to eat according the Dutch recommended dietary guidelines [20]. These instructions were repeated at every visit throughout the study to optimize adherence. A validated food frequency questionnaire (FFQ) was completed at the end of both periods to assess energy and nutrient intake during the preceding month. For these calculations we used the Dutch food composition table (NEVO Table; [21]).

**Fig. 1:**
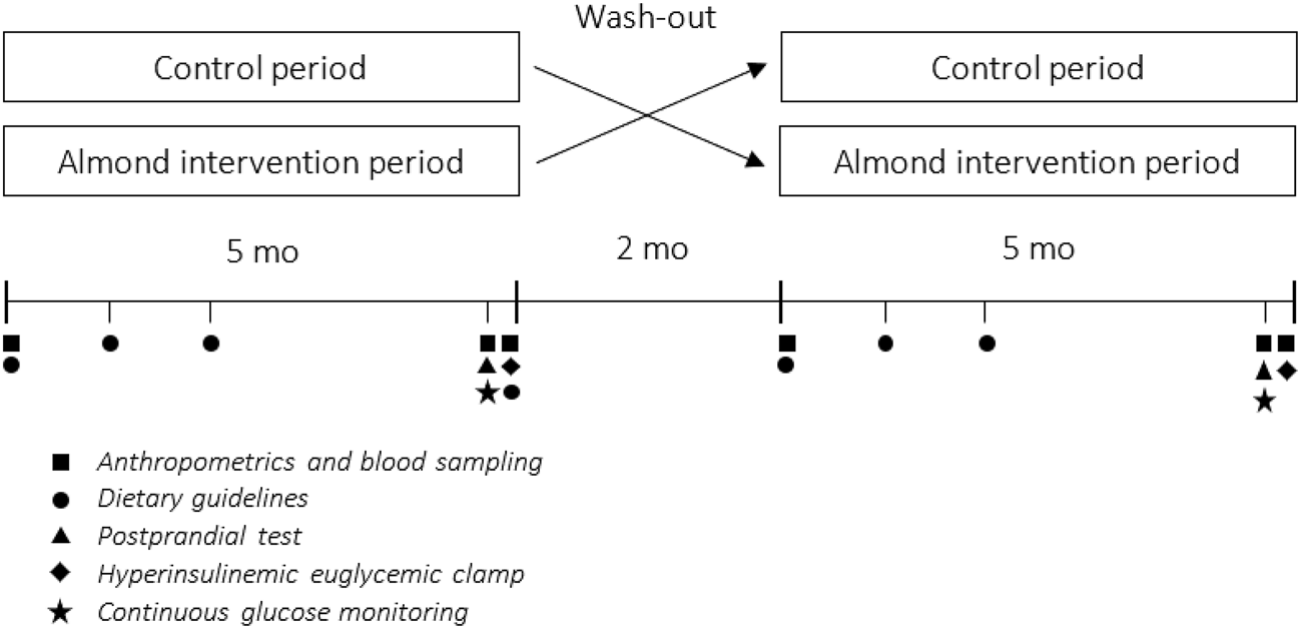
Study design

A visit was planned at the start of both the intervention and control period for quantifying anthropometrics and blood pressure, and blood sampling. Next, two test days were scheduled at the end of each period, *i.e.,* one test day for quantifying anthropometrics and blood pressure, performing a postprandial test and placement of the continuous glucose sensor, and the other test day again for quantifying anthropometrics and blood pressure, and performing a hyperinsulinemic euglycemic clamp (Fig. 1). Participants were asked to abstain from strenuous physical activity and alcohol consumption two days prior to the test days. They consumed a standardized, commercially available, high-carbohydrate meal and dessert (829 kcal, 10.1 percent of energy (En%) fat, 75.0 En% carbohydrates, and 15.0 En% protein) at a fixed time point the evening preceding both test days to eliminate potential effects of the previous meal. The next morning, they arrived at the MRUM research facilities by car or public transport after a 12-h overnight fast (no food or drinks after 8 PM, except water).

### Anthropometrics and blood pressure

Upon arrival at each test day, height, body weight, and waist and hip circumference were measured. While participants were seated, systolic and diastolic blood pressure, and heart rate were measured after a 10-min rest with an intermittent blood pressure monitoring device (Omron Intellisense M7; Cemex Medische Techniek, Nieuwegein, the Netherlands). Blood pressure measurements were repeated at least four times, of which the first measurement was discarded, and averaged over the three final measurements.

### Hyperinsulinemic euglycemic clamp

To measure whole-body insulin sensitivity, a 1-step hyperinsulinemic euglycemic clamp was performed as described by DeFronzo et al*.* [18]. One cannula was inserted in the antecubital vein for co-infusion of insulin (40 mU/m^2^/min; Novorapid, Novo Nordisk, Bagsværd, Denmark) and a 20% glucose solution. A second cannula in the other arm was used for collecting a fasting blood sample, amongst others for acute analyses of K and Hb, and continued to be used for venous blood sampling during the clamp. With the start of the clamp, participants received a 10-min prime of insulin infusion (60 mU/m^2^/min). Throughout the entire clamp, every 5–10 min glucose concentrations were measured directly. The glucose infusion rate (GIR) was adjusted to maintain euglycemia at 5.0 mmol/L and reach a 20-min steady-state condition. The GIR was used to calculate the M-value (mg/kg/min) to determine insulin sensitivity as described earlier [18].

### Postprandial test

For the postprandial test, a cannula was inserted in the antecubital vein for blood sampling. After a fasting blood sample was collected (T0), participants received a high-fat mixed meal in the form of a shake, which they had to consume within 10 min. An independent researcher freshly prepared the shakes on the morning of the postprandial test day. The shake consisted of 53.0 En% from fat, 36.4 En% from carbohydrates (mainly simple carbohydrates), and 11.9 En% from protein, with an overall energy content of 711 kcal. Postprandial blood samples were collected at 15 min (T15), 30 min (T30), 45 min (T45), 60 min (T60), 90 min (T90), 120 min (T120), 180 min (T180), and 240 min (T240) after shake consumption.

### Continuous glucose monitoring

Between the two test days, glucose profiles were monitored continuously (CGM) using a Freestyle Libre Pro sensor (Abbott, Alameda, California, United States). The sensor was applied after completion of the first test day and profiles were recorded for 48 h, starting at 12 AM. The sensor was placed on the back of the upper arm to measure glucose concentrations every 15 min. The moving average was calculated for every 15 min over the preceding hour. These values were used to calculate the AUC and iAUCmin per 24 h with GraphPad Prism 8, and averaged between the two days. As baseline for the iAUCmin, the minimum moving average for each 24-h period was used. Furthermore, the mean 24-h maximum moving average of the continuous glucose concentrations was calculated.

### Biochemical analyses

At the start of each period and on the postprandial and clamp test day fasting blood was sampled in 3.5 mL serum ST-II advance tubes and 2 mL sodium fluoride (NaF) and Na_2_EDTA containing tubes (Becton Dickinson). A minimum clotting time of 30 min at room temperature was applied to the serum tubes. Serum samples were then centrifuged at 1300 × g for 10 min at 21 °C, whereas NaF samples were centrifuged at 1300 × g for 10 min at 4 °C within 30 min after sampling. Following centrifugation, serum and plasma were portioned into aliquots, frozen into liquid nitrogen, and stored at -80 °C until biochemical analyses upon study completion. Serum samples were used for the analyses of TC (CHOD-PAP method; Roche Diagnostics, Mannheim, Germany), HDL cholesterol (precipitation method, Roche Diagnostics, Mannheim, Germany), TAG (GPO Trinder, Sigma-Aldrich Corp., St. Louis, MO, United States) with correction for free glycerol, and insulin (human insulin specific RIA kit, Millipore, Billerica, MA, United States) concentrations. Plasma samples were used for the analyses of glucose (Glucose HK CP, Horiba ABX, Montpellier, France) concentrations. LDL cholesterol concentrations were calculated using the Friedewald formula [22], and the homeostatic model assessment for insulin resistance (HOMA-IR) was calculated using the formula: fasting insulin (μU/mL) x fasting glucose (mmol/L)/22.5 [23].

### Statistical analysis

Data are presented as mean ± standard deviation (SD), unless stated otherwise. Based on a power calculation before the start of the study, 34 participants were needed to detect a true difference of 15% in insulin sensitivity expressed as the M-value with a within-subject variability of 4.23%, a power of 80%, and a two-tailed alpha of 0.05.

Values at the start of each period were compared with a paired-samples t-test. Differences in BMI and age between prediabetic states, *i.e.* IFG, IGT, or both, were analyzed with a one-way ANOVA. Differences in anthropometrics, fasting concentrations, and postprandial responses between the control and intervention period were analyzed with linear mixed models. The anthropometric and biochemical values at the end of the control and intervention period were used as dependent variable with subject as random factor, with randomization order, period, and treatment as fixed factors, and with baseline values as covariate. The same linear mixed models were used with the absolute postprandial glucose and insulin concentrations as dependent variables with subject added as random factor, and time and the interaction term [treatment*time] as fixed factors, with fasting glucose and insulin concentrations, and BMI as covariate. If the randomization order and/or interaction term was not significant, it was omitted from the model. If the interaction term was significant, concentrations between treatments were analyzed per time point with Bonferroni adjustment for multiple comparisons. Another linear mixed model was used to examine differences in FFQ, clamp, postprandial AUC, and CGM data between the control and intervention periods which were only measured at the end of both periods. Subject was used as random factor, and randomization order, period, and treatment were used as fixed factors, and BMI was used as covariate, except for the FFQ data. If the randomization order was not significant, it was omitted from the model, indicating there was no carry-over effect. For all tests, two-sided p-values ≤ 0.05 were considered statistically significant and analyses were performed using IBM SPSS Statistics for Windows, Version 26.0 (IBM Corporation, Armonk, NY, United States).

## Results

### Study participants and compliance

**Fig. 2:**
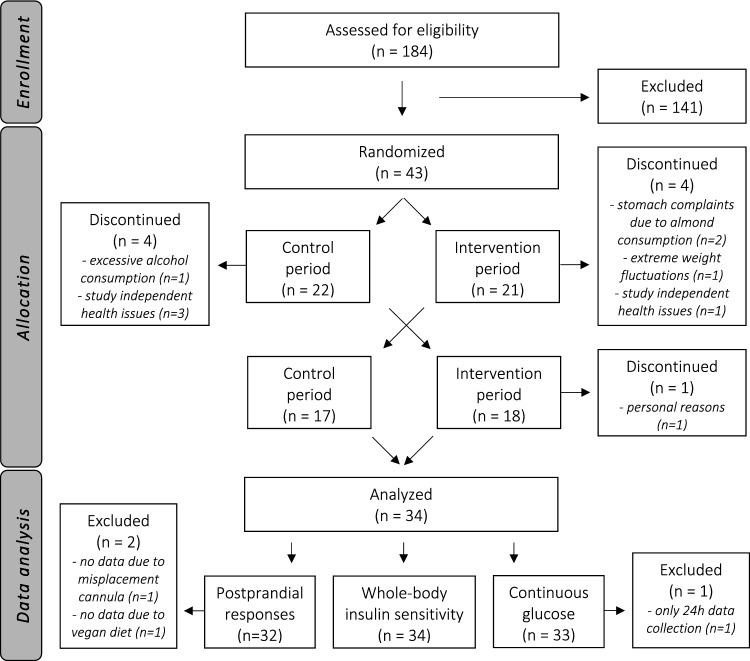
Flowchart of participants

One hundred eighty-four males and females were assessed for eligibility via a screening visit. As shown in the flowchart, 43 participants were eventually included and randomized to start either with the almond or control period (Fig. 2). Nine participants retracted informed consent or were withdrawn from the study by the researcher. Participants discontinued the study due to stomach complaints possibly related to the almond consumption (n = 2), study independent health issues (n = 4), excessive alcohol consumption (n = 1), extreme weight fluctuations (n = 1), or personal reasons (n = 1). In total, 34 participants completed the study. Clamp data was available in all 34 participants. However, postprandial data were missing from two participants, which means that data from 32 participants were used for postprandial analyses. The reason was a misplacement of the cannula on the postprandial test day (n = 1) or commitment to a vegan diet, which did not match with the standardized high-fat mixed meal (n = 1). Furthermore, one participant was excluded from the continuous glucose analyses as only a 24 h profile was collected, which resulted in data of 33 participants for CGM analyses. Finally, FFQ data were missing from two participants, which means that data from 32 participants were used for FFQ analyses.

The group that completed the study consisted of 22 males (65%) and 12 females (35%) with the following prediabetes status: 10 IFG (29%), 12 IGT (35%), and 12 IFG plus IGT (35%). They had a median (25–75th percentiles) age of 66 (56–69) years and BMI of 28.3 (26.8–33.7) kg/m^2^, which did not significantly differ between prediabetes status (P = 0.807 and P = 0.470, respectively). Participant characteristics that were measured on the two screening visits before the start of the study are presented in Table 1. Based on accountability logs and diaries, participants had a mean compliance of 98.0 ± 2.8% during the intervention period.

**Table 1:** Participant characteristics

Characteristic	n = 34
Males/females (%)	65/35
IFG/IGT/IFG & IGT (n)	10/12/12
Age (years)	66 (56–69)
BMI (kg/m^2^)	28.3 (26.8–33.7)
Waist circumference (cm)	102.6 ± 11.7
Waist-to-hip ratio	0.98 ± 0.08
Systolic BP (mmHg)	126 ± 15
Diastolic BP (mmHg)	79 ± 9
Heart rate (BPM)	67 (60–76)
Glucose (mmol/L)	6.08 ± 0.60
2-h glucose (mmol/L)	8.36 ± 1.92
Total cholesterol (mmol/L)	5.40 ± 0.95
TAG (mmol/L)	1.33 (1.06–1.87)

Values are presented as mean ± SD or median with ranges (25–5th percentiles); *IFG* impaired fasting glucose, *IGT* impaired glucose tolerance, *BMI* body mass index, *BP* blood pressure, *TAG* triacylglycerol

### Anthropometrics, biochemical parameters, and dietary composition during the study

Anthropometrics and biochemical parameters were not significantly different at the start of both periods. The final statistical model to evaluate the effect of almond consumption on anthropometrics and biochemical parameters contained treatment and period. As shown in Table 2, BMI (P = 0.002), waist circumference (P = 0.013), and fasting insulin concentrations (P = 0.003) were significantly higher after consuming 50 g almonds daily for five months compared to the control period. The remaining anthropometric and fasting biochemical parameters did not significantly change after almond consumption as compared to control.

**Table 2:** Mean anthropometric and biochemical characteristics at baseline and at the end of control and intervention period (n = 34)

	Baseline control	End control	Baseline intervention	End intervention	Treatment effect (95% CI); *P* value
BMI (kg/m^2^)	29.9 ± 4.2	29.6 ± 4.6	29.8 ± 4.5	30.2 ± 4.5	+ 0.6 (0.2, 1.0); 0.002*
Waist circumference (cm)	103.2 ± 11.9	101.9 ± 12.2	102.7 ± 13.4	103.9 ± 11.1	+ 2.4 (0.5, 4.3); 0.013*
Waist-to-hip ratio	0.98 ± 0.07	0.99 ± 0.06	0.99 ± 0.08	1.00 ± 0.05	+ 0.01 (– 0.01, 0.02); 0.385
Systolic BP (mmHg)	130 ± 17	128 ± 15	128 ± 15	129 ± 15	+ 3 (– 1, 6); 0.154
Diastolic BP (mmHg)	83 ± 9	80 ± 8	82 ± 10	81 ± 7	+ 1 (– 1, 3); 0.428
Heart rate (BPM)	65 ± 10	63 ± 9	63 ± 8	63 ± 9	+ 1 (– 2, 4); 0.599
Fasting glucose (mmol/L)	6.35 ± 0.68	6.27 ± 1.01	6.36 ± 0.85	6.27 ± 0.86	– 0.02 (– 0.25, 0.21); 0.862
Fasting total cholesterol (mmol/L)	5.71 ± 1.05	5.26 ± 0.89	5.69 ± 1.08	5.06 ± 0.86	– 0.19 (– 0.43, 0.05); 0.116
Fasting HDL cholesterol (mmol/L)	1.17 ± 0.27	1.10 ± 0.27	1.19 ± 0.29	1.04 ± 0.27	– 0.07 (– 0.14, 0.01); 0.068
Fasting LDL cholesterol (mmol/L)	3.80 ± 0.93	3.42 ± 0.72	3.79 ± 0.89	3.22 ± 0.75	– 0.18 (– 0.38, 0.01); 0.063
Fasting TAG (mmol/L)	1.60 ± 0.80	1.64 ± 0.71	1.54 ± 0.80	1.73 ± 0.89	+ 0.15 (– 0.05, 0.35); 0.131
Fasting insulin (μU/mL)	18.30 ± 10.10	15.16 ± 9.88	16.78 ± 10.39	16.55 ± 9.62	+ 2.35 (0.85, 3.85); 0.003*
HOMA-IR	5.25 ± 3.16	4.52 ± 4.12	4.90 ± 3.54	4.78 ± 3.21	+ 0.52 (– 0.09, 1.14); 0.091

Values are presented as mean ± SD; *significant difference in the change from baseline values between control and intervention (P < 0.05); *IFG* impaired fasting glucose, *IGT* impaired glucose tolerance, *BMI* body mass index, *BP* blood pressure, *HDL* high-density lipoprotein, *LDL* low-density lipoprotein, *TAG* triacylglycerol, *HOMA-IR* homeostatic model assessment for insulin resistance

Data obtained from the FFQ indicated that during the almond intervention period participants had a higher energy intake (P = 0.016), and a higher En% intake of total fat (P < 0.001), monounsaturated fatty acids (MUFA; P < 0.001), oleic acid (P < 0.001), polyunsaturated fatty acids (PUFA; P < 0.001), linoleic acid (P < 0.001), eicosapentaenoic acid (EPA; P = 0.017), docosahexaenoic acid (DHA; P = 0.015), and fiber (P = 0.002) compared to the control period, whereas intake of saturated fatty acids (SFA; P = 0.044), alpha-linolenic acid (ALA; P = 0.049), and total carbohydrates (P = 0.009) were lower (Table 3).

**Table 3:** Dietary composition calculated over the last month of the control and intervention period (n = 32)

Nutritional value	Control	Intervention	Difference (95% CI); *P* value
Energy (kcal)	2146 ± 565	2299 ± 460	+ 162 (32, 292); 0.016*
Total fat (En%)	37.1 ± 4.3	43.2 ± 4.1	+ 6 (4.8, 7.3); < 0.001**
SFA (En%)	12.0 ± 3.5	11.3 ± 3.2	– 0.8 (– 1.6, 0.0); 0.044*
MUFA (En%)	13.6 ± 2.3	18.5 ± 2.8	+ 4.8 (4.1, 5.5); < 0.001**
Oleic acid (En%)	11.3 ± 2.4	16.7 ± 2.9	+ 5.3 (4.4, 6.3); < 0.001**
PUFA (En%)	7.8 ± 2.1	9.6 ± 2.0	+ 1.8 (1.2, 2.4); < 0.001**
Linoleic acid (En%)	6.3 ± 1.7	8.3 ± 1.7	+ 2.0 (1.5, 2.5); < 0.001**
ALA (En%)	0.83 ± 0.33	0.75 ± 0.28	– 0.08 (– 0.16, 0.00); 0.049*
EPA (En%)	0.04 ± 0.03	0.06 ± 0.05	+ 0.02 (0.00, 0.04); 0.017*
DHA (En%)	0.07 ± 0.05	0.10 ± 0.08	+ 0.03 (0.01, 0.06); 0.015*
Total carbohydrates (En%)	41.9 ± 4.5	39.9 ± 4.1	– 2.0 (– 3.5, – 0.5); 0.009*
Fiber (En%)	2.5 ± 0.7	2.8 ± 0.5	+ 0.3 (0.1, 0.5); 0.002*
Fiber (g)	27.3 ± 9.7	32.4 ± 5.9	+ 5.3 (2.9, 7.8); < 0.001**
Total protein (En%)	16.5 ± 2.3	16.8 ± 2.1	+ 0.4 (– 0.2, 1.1); 0.178

Values are presented as mean ± SD; *significant difference between control and intervention (P < 0.05); ** significant difference between control and intervention (P < 0.001); *SFA* saturated fatty acids, *MUFA* monounsaturated fatty acids, *PUFA* polyunsaturated fatty acids, *ALA* alpha-linolenic acid, *EPA* eicosapentaenoic acid, *DHA* docosahexaenoic acid

### Effect of almond consumption on insulin sensitivity

**Fig. 3:**
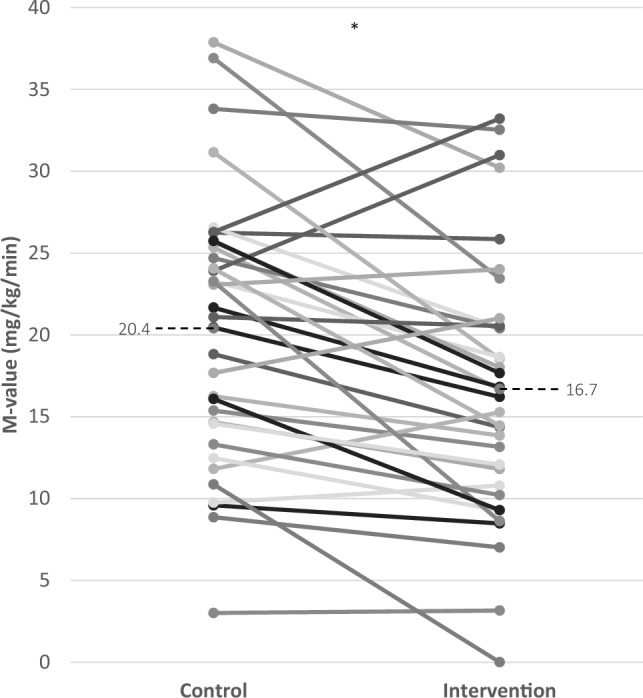
The change in M-value (mg/kg/min) between the control and intervention period for each participant, including the average M-value of the control (20.4 ± 8.2 mg/kg/min) and intervention period (16.7 ± 8.0 mg/kg/min; dotted line)

The final statistical model to evaluate the effect of almonds on insulin sensitivity as measured by the hyperinsulinemic euglycemic clamp contained period, and treatment, with BMI as covariate. Whole-body insulin sensitivity expressed as the M-value was significantly lower at the end of the almond intervention period (16.7 ± 8.0 mg/kg/min) compared to the control period (20.4 ± 8.2 mg/kg/min; P = 0.002). The treatment effect of almonds on the M-value was estimated at + 3.15 mg/kg/min (95% CI 1.28, 5.01), whereas the same model without BMI as covariate estimated the treatment effect at + 3.78 mg/kg/min (95% CI 1.94, 5.63; P < 0.001). This indicates that the change in BMI only partly explained the treatment effect on the M-value. The individual changes in M-value are shown in Fig. 3. In line with the M-value, the HOMA-IR was higher after the almond intervention period compared to the control period, but this did not reach statistical significance (P = 0.091; Table 2).

### Effect of almond consumption on postprandial glucose metabolism

**Fig. 4:**
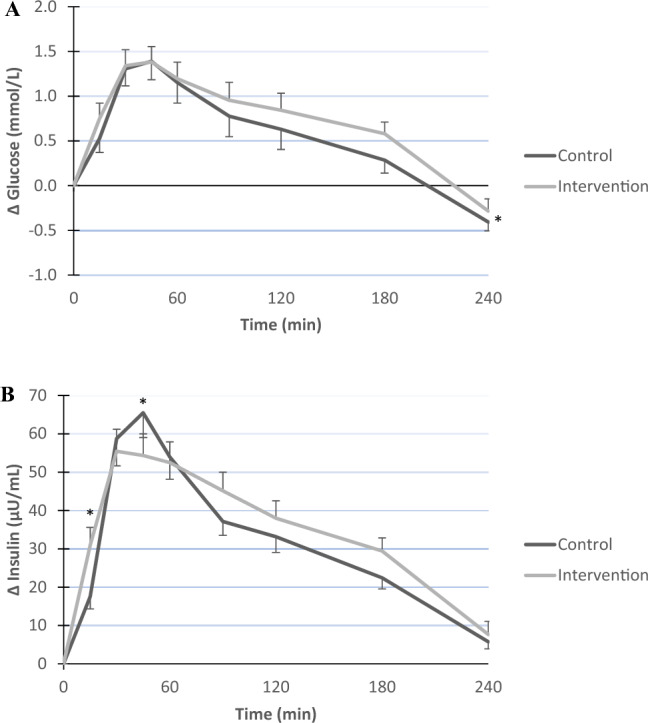
Postprandial mean changes (± SE) in glucose (mmol/L; **A**) and insulin concentrations (μU/mL; **B**) after the control (black line) and intervention period (gray line)

The final statistical model to evaluate the effect of almonds on postprandial glucose responses contained period, time, and treatment. Besides randomization order, the interaction term [treatment*time] was omitted from the model (P = 0.597), indicating that the changes over time did not significantly depend on treatment. The postprandial glucose response curve was significantly different between the control and intervention period (P = 0.019; Fig. 4A). In more detail, the total AUC based on the postprandial glucose concentrations was significantly higher at the end of the almond intervention period (1654 ± 193 mmol/L/240 min) as compared to the control period (1601 ± 209 mmol/L/240 min; P = 0.040). Finally, the iAUC for glucose was also higher after the almond intervention period compared to the control period, but did not reach statistical significance (P = 0.089).

The final statistical model to evaluate the effect of almonds on postprandial insulin responses contained period, time, treatment, and the interaction term [treatment*time] (P = 0.006). The postprandial insulin response curve was not significantly different between the control and intervention period (P = 0.679; Fig. 4B). More specific, the treatment effects were significant at time point T15 (P = 0.022) and T45 (P = 0.025). The total AUC based on the postprandial insulin concentrations was not significantly different at the end of the almond intervention period (11,902 ± 4479 μU/mg/240 min) as compared to the control period (10,572 ± 3927 μU/mg/240 min; P = 0.080). The iAUC for insulin was not significantly different between the two experimental periods (P = 0.277).

### Effect of almond consumption on 48 h continuous glucose concentrations

**Fig. 5:**
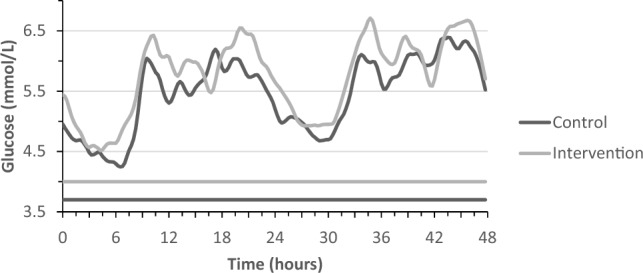
Mean moving average 48 h continuous glucose concentrations after the control (black line) and intervention period (gray line). The horizontal lines represent the mean moving average minimum glucose concentration for the control (3.72 ± 0.91 mmol/L) and intervention period (3.98 ± 0.82 mmol/L)

The final statistical model to evaluate the effect of almonds on continuous glucose concentrations contained period, and treatment. The 48 h continuously monitored glucose concentrations of both periods are shown in Fig. 5. The total AUC for the 24 h continuous glucose profiles did not significantly differ between the almond intervention period (8191 ± 1539 mmol/L/24 h) and the control period (7789 ± 1595 mmol/L/24 h; P = 0.066). The iAUCmin did not significantly differ between the almond intervention period (2528 ± 902 mmol/L/24 h) and the control period (2494 ± 789 mmol/L/24 h; P = 0.926). Furthermore, the minimum 24 h glucose concentration was significantly higher after the almond intervention (3.98 ± 0.82 mmol/L) as compared to the control period (3.72 ± 0.91 mmol/L; P = 0.038), whereas the maximum 24 h glucose concentration after the almond intervention was 8.75 ± 1.47 mmol/L as compared to 8.40 ± 1.75 mmol/L after the control period (P = 0.154).

## Discussion

The aim of this randomized controlled trial was to examine the effects of long-term almond consumption on insulin sensitivity and glucose metabolism in males and females with prediabetes. So far, the hypoglycaemic effects of almonds are inconsistent [10, 11, 13–16]. We decided here to examine the effects of almonds in a free-living setting without detailed instructions how to incorporate the almonds in the habitual diet. Effects were evaluated by different approaches on insulin sensitivity and glucose metabolism side-by-side. The results showed a significantly lowered whole-body insulin sensitivity as measured by the hyperinsulinemic euglycemic clamp and increased postprandial glucose responses after the almond intervention. The AUC for 24 h continuous glucose concentrations did not reach statistical significance, but fasting insulin concentrations were also increased. The significantly increased BMI, waist circumference, and energy intake after the almond period implied that almonds were added to the habitual diet and did not fully replace other foods. Since the estimated treatment effect for almonds on the M-value with and without correction for changes in BMI were nearly identical, it must be concluded that the BMI increase could only partly explain the adverse effect on insulin sensitivity.

A major question is how the inconsistent results regarding possible hypoglycaemic effects of almonds as presented in the literature [10, 11, 13–16] can be explained. First, there is a possibility that the discrepant findings are related to duration of the intervention period. No effects were found after daily consumption of 100 g almonds for 4 weeks [16], 85 g for 6 weeks [13], 20% of daily estimated energy requirements for 6 weeks [15], or 56 g for 12 weeks [14]. The studies with beneficial effects were found after 57 g almond consumption for 16 weeks [11], or already after daily 57 g almond consumption for 4 weeks [10]. Therefore, it seems that study duration is not the reason for the inconsistent study findings and it seems unlikely that our findings can be ascribed to this. Second, there is a possibility that the discrepant findings are related to the amounts of almonds consumed. The studies that showed beneficial effects [10, 11] used a comparable or lower amount (57 g) than our study and the studies that did not show an effect, ranging between 50 and 100 g [13–15]. Therefore, it seems that the amounts of almonds consumed is not the reason for the inconsistent study findings.

Besides those two options, we postulated that changes in body weight could be an explanation for the inconsistent results. In line with Lovejoy et al*.* [16], we found an increase in body weight and speculated that this could be the reason that we did not find a beneficial almond effect in our study. Almonds presumably increase energy intake, but based on a meta-analysis, diets enriched in nuts did not result in weight gain [24]. However, the estimated effects of nut consumption were based on studies that imposed energy restriction or weight maintenance. In controlled feeding studies, no weight changes were found when intervention and control aimed to be isoenergetic or when participants received instructions on the incorporation of food substitutions [13–15]. However, as described earlier, participants increased energy intake and body weight during almond supplementation, despite advice on food substitutions was provided [16]. That study did not find an effect of almonds on glucose profiles, which could be related to body weight increases. There is a well-known link between weight and insulin sensitivity as weight loss improved insulin resistance [25], and weight is negatively correlated with M-value in IGT individuals [26]. Weight gain exacerbates insulin resistance as excess body fat, particularly visceral fat, increases the release of free fatty acids, hormones, and proinflammatory substances [27]. On the one hand, some of these substances contribute to energy balance and glucose homeostasis. On the other hand, they may induce an inflammatory state and oxidative stress, all leading to insulin resistance [28]. However, the negative effect of almonds on insulin sensitivity as we describe here can only partly be explained by the increase in body weight, because after adjusting for changes in BMI in the statistical model, the negative effect of almonds largely remained. The potential effects of increased almond consumption on body weight requires attention since weight gain related to a dietary advice cannot be definitively interpreted. Aside from the dietary recommendation to consume nuts for cardiovascular prevention [7], the guidelines might not provide sufficient guidance regarding how to adjust other factors determining energy balance, as mimicked in the current study. Therefore, to our opinion, it is important to emphasize more how energy-dense foods like nuts should be incorporated in the healthy diet.

As the adverse effects of long-term almond consumption cannot only be ascribed to body weight, we need to examine other possible explanations. Considering the nutrient composition of almonds, it is surprising that we found these adverse effects on glucose metabolism. Theoretically, it could relate to the overfeeding of fats due to the almond consumption, resulting in fat accumulation in the liver [29]. It is well established that liver fat accumulation is associated with insulin resistance [30]. However, almonds specifically contain a high amount of PUFA, which seems to prevent liver fat accumulation compared to SFA overfeeding [31], which excludes this explanation. Furthermore, we could speculate that the acrylamide content found in processed almonds contribute to the lack of health effects [32]. Acrylamide is naturally formed by a chemical reaction between the amino acid asparagine and reducing sugars under high temperatures [33]. Since almonds are rich in these precursors, they are prone to acrylamide formation [34]. Although acrylamide is considered an unfavorable compound, it seems to be negatively associated with insulin resistance [35] and glucose concentrations [36]. However, acrylamide can only be found in roasted almonds, whereas participants in the current study consumed natural whole almonds. Other compounds such as advanced glycation end products (AGEs) could also contribute to the adverse effects found as they are linked to insulin resistance [37]. Although AGEs can be found in both roasted and raw almonds, they were significantly increased in roasted almonds as compared to raw almonds [38].

As said, already a number of studies could not show a beneficial effect of almonds on insulin sensitivity and glucose metabolism. To the best of our knowledge, we are the first study that showed adverse effects upon almond consumption. Though unexpected, findings are consistent between the different approaches used, *i.e.*, the hyperinsulinemic euglycemic clamp, the postprandial test, and fasting insulin concentrations. Moreover, changes in 48 h continuous glucose monitoring and HOMA-IR did not reach statistical significance but showed a trend towards an increase after almond consumption compared to control. This finding for the HOMA-IR being not significant might be explained by the suggestion that HOMA-IR is a better reflection of hepatic insulin sensitivity, whereas the clamp is a better reflection of peripheral insulin sensitivity [39]. This would imply that almonds preferentially affect peripheral insulin sensitivity. More likely is that HOMA-IR is reflecting the temporary insulin sensitive state compared to the well-controlled M-value by the clamp. For example, glucose concentrations that are used to calculate HOMA-IR are more influenced by an extended fasting duration [40] than in the insulin-stimulated clamp.

In conclusion, our findings suggest that long-term almond consumption may negatively affect insulin sensitivity and glucose metabolism in subjects with prediabetes. These adverse effects could not be explained by the observed increase in body weight alone. Nevertheless, almonds have been consistently shown to positively affect other cardiometabolic risk factors [41]. In real life conditions, it might be necessary to provide more supporting guidelines on how to incorporate energy-dense foods like nuts into healthy diets to prevent weight gain and its consequences. Further research should focus on the added value of nutrient-dense almonds in preventing type 2 diabetes.

## Data Availability

Data described in the manuscript, code book, and analytic code will be made available upon request from the first author.

## Abbreviations

AGE: Advanced glycation end product
ALA: Alpha-linolenic acid
BMI: Body mass index
CGM: Continuous glucose monitoring
DHA: Docosahexaenoic acid
EPA: Eicosapentaenoic acid
FFQ: Food frequency questionnaire
GIR: Glucose infusion rate
Hb: Haemoglobin
HOMA-IR: Homeostatic model assessment for insulin resistance
IFG: Impaired fasting glucose
IGT: Impaired glucose tolerance
K: Potassium
MUFA: Monounsaturated fatty acids
NaF: Sodium fluoride
OGTT: Oral glucose tolerance test
PUFA: Polyunsaturated fatty acids
SFA: Saturated fatty acids
TAG: Triacylglycerol
TC: Total cholesterol
